# Perinatal outcomes of reduced fetal movements: a cohort study

**DOI:** 10.1186/s12884-016-0964-2

**Published:** 2016-07-19

**Authors:** Claire M. McCarthy, S. Meaney, K. O’Donoghue

**Affiliations:** Department of Obstetrics and Gynaecology, Cork University Maternity Hospital, University College Cork, Wilton, Cork, Ireland; National Perinatal Epidemiology Centre, University College Cork, Cork, Ireland

**Keywords:** Fetal movement, Perinatal outcome, Stillbirth, Kick counting

## Abstract

**Background:**

The perception of reduced fetal movement (RFM) is an important marker of fetal wellbeing and is associated with poor perinatal outcome (such as intra-uterine death).

**Methods:**

We conducted a prospective study of women presenting with RFM over 28 weeks’ gestation to a tertiary-level maternity hospital. We examined pregnancy outcomes and compared them to a retrospectively collected control group delivering contemporaneously.

**Results:**

In total, 275 presentations were analysed in the RFM group, with 264 in the control group. Women with RFM were more likely to be nulliparous (*p* = 0.002) and have an induction of labour (*p* = 0.0011). 26.5 % (*n* = 73) of cases were admitted following presentation with RFM, and 79.4 % (*n* = 58) delivered on primary presentation. Overall, 15.2 % (*n* = 42) women were induced for RFM specifically.

**Conclusion:**

This prospective study shows the increased burden of care required by those with RFM, including increased neonatal unit admission rates, increased induction rates and higher surveillance demands, demonstrating the need for increased attention to this area of practice.

## Background

Maternal perception of reduced fetal movements (RFM) in pregnancy is a common reason for self-referral for assessment by healthcare providers in pregnancy [1]. RFM has been shown to occur in up to 15 % of pregnancies, and comprises of 6.1 % of the workload of acute maternity assessment services [1]. It has been found that up to 55 % of women who have a stillbirth note a reduction in fetal movement prior to diagnosis [2]. It has also been hypothesized that inadequate clinician response to the complaint of RFM is an important contributory factor to stillbirth [3]. Factors relating to sub-optimal care are implicated in up to 50 % of stillbirths, and suboptimal care is continually highlighted in international maternal morbidity and mortality enquiries [4, 5].

Fetal movement counting is a long-standing method of assessing fetal wellbeing [6]. Froen et al have reported that those presenting with RFM in pregnancy had an increased perinatal mortality, increased need for emergency delivery and low neonatal Apgar scores at delivery [3, 7]. International guidelines also note disparities in the definition and management of RFM [8–10]. There is no evidence that any formal definition of RFM is of greater value than the subjective maternal perception in the detection of fetal compromise [11]. There are no current randomised trials comparing pregnancy outcomes in those who employed fetal movement counting and those who did not utilise formal fetal movement counting, and therefore there  is little consensus to advise clinical practice in the area of RFM [6]. The implementation of formal counting strategies is increasingly a controversial issue, with some studies demonstrating no significant benefit to fetal movement counting [12]. Conversely, some studies have shown that fetal movement counting reduces perinatal mortality, with little economic impact [13]. It has been noted that consensus on the management of RFM is difficult to achieve due to lack of definitive guidance [14].

Further, more recent studies have recommended protocols to assess and manage RFM, and along with patient education, have decreased their institutional stillbirth rate from 4.2 % to 2.4 % in women with RFM [15].

From review of the literature, it is clear that there are a limited number of large scale studies comparing outcomes of pregnancies affected by RFM, particularly in the Irish context. As RFM is a significant precursor to intra-uterine fetal death, attention needs to be focussed on related areas of intervention.

The primary objective of this observational study was to assess the pregnancy characteristics and outcomes of pregnant women presenting to hospital with RFM. We examined the incidence and demographics of these women, and evaluated their investigation and management pathways.

## Methods

We conducted a cohort study, prospectively recruiting all women presenting to the Emergency Department (ED) of a large university-based tertiary referral maternity hospital (with over 8000 deliveries per annum) with a complaint of RFM from 1st April 2013 until 31st October 2013. We included women over 28 weeks’ gestation, presenting with RFM, and delivering in our hospital during the study period. We included women booking at both low and high risk antenatal clinics. Exclusion criteria were women with multiple pregnancy and/or pregnancies with antenatally-diagnosed congenital anomalies. During data collection, medical conditions and medication taken at the time of booking were recorded. Medications prescribed during the pregnancy were not recorded as the primary aim of our study was not to establish a relationship between medical administration and RFM.

The ED is a 24-h service, assessing approximately 17,500 women per year, staffed by midwives and a senior house officer, with registrar support. There is ultrasound unit support, with increased staffing between the hours of 0800 and 1700. Women were recruited on presentation to the ED from the attendance logbook. Information obtained at this stage included time of presentation, duration of time in the ED and outcome of visit. Pregnancy characteristics and outcomes were assessed by the first author (CM) following delivery by chart review.

Data were collected on demographics (e.g. age and marital status), pregnancy related characteristics (e.g. parity, conception and gestation) and a number of risk factors for intrauterine fetal death and fetal growth restriction (e.g. smoking, previous pregnancy loss and obesity). The antenatal presentation was examined, and included modes of evaluation of RFM, including blood pressure and assessment for hypertension (classified as more than 140/90 mmHg), Cardiotocograph (CTG), ultrasound assessment and formal departmental ultrasound assessment. Finally, delivery (e.g. onset of labour, mode of delivery and Apgar scores) and postpartum (e.g. neonatal admission) information was obtained. Due to the inaccessibility of some charts collected for both groups, full demographic data were not available for several women. Missing data were subsequently sought from delivery logbooks and birth registers, however some variables were irretrievable. The most common missing variable was infant birth weight at delivery (20/275) and maternal body mass index (10/275). High-risk patients accounted for 17.1 % (47/275) of the total RFM cohort, however due to a large variety of reasons for referral to the Perinatal Medicine Clinic, reason for classification as a high-risk pregnancy was not recorded.

Women with RFM were compared to a control sample comprising of women who delivered contemporaneously. These women were randomly sampled, retrospectively, from a database of all women who delivered an infant during the study period. Sampling involved systematically selecting every 14th entry from the data base, which included both low and high-risk pregnancies. Relevant information was then obtained from the delivery records stored on the delivery suite. These maternity charts were not available for review as charts of women who have delivered are transported to a storage facility off-site following delivery; therefore, limited characteristics were available for comparison (namely age, gravidity, parity, onset of labour, mode of delivery and neonatal outcomes).

Data analysis was performed with PASW Statistics (PASW Statistics 18, IBM, 2009) and Microsoft Excel (Microsoft Excel, 2013). Continuous data were analysed using the *t*-test, and categorical data were analysed using Pearson’s Chi-squared test.

## Results

### Baseline characteristics and demographics

**Fig. 1:**
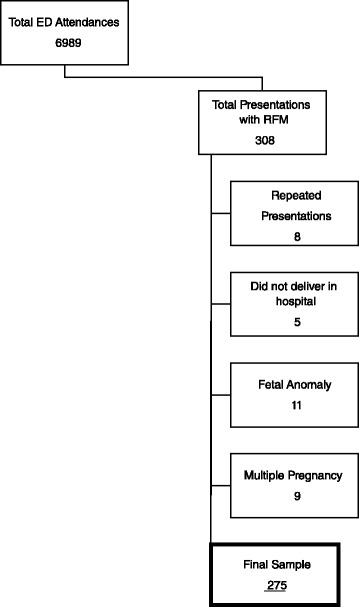
Flow diagram with exclusion criteria

As outlined in Fig. 1 there were 6989 attendances (including antenatal, postnatal, early pregnancy and gynaecology cases) to the ED during the study period, with 308 (4.4 %) presentations pertaining to RFM. Of these, 8 women presented a second time with RFM. A further 5 women were excluded as they did not deliver in our hospital. Additionally, 11 women with a known fetal anomaly and 9 women with multiple pregnancy were excluded. Therefore, our final sample consisted of 275 pregnant women, with the control group comprising 265 women.

On average, 39 women presented with RFM to the ED each month, corresponding to just over one presentation with RFM per day. Of all the cases, 1.5 % (*n* = 4) of women were diagnosed with an intra-uterine fetal death in their pregnancy on presentation, correlating to a 14.5 per 1000 stillbirth rate in the group of women presenting over 28 weeks’ gestation with RFM.

Table 1 demonstrates baseline characteristics and demographics of women in both groups. The majority of women in both groups were under 35 years of age (69.5 % and 69.8 %). Of those in the RFM group, 23 % (*n* = 63) of women had a body mass index over 30 kg/m^2^, and 85 % (*n* = 232) were non-smokers. Nearly one third of women had a previous first trimester miscarriage (28.2 %; *n* = 76). Half of women in the RFM group were primiparous (50.2 %; *n* = 138) compared to a third of women in the control group (37 %; *n* = 98). Over three quarters of women described their nationality as Irish (78.5 %; *n* = 216).

**Table 1:** Baseline characteristics and demographics of RFM group

Characteristic	RFM Group % (*n*)
Age	
Under 35 years	69.5 (191)
Over 35 years	30.5 (84)
Marital Status	
Single	32 (88)
Married	65.5 (180)
Divorced/Separated	1.1 (3)
Not reported/Missing	1.5 (4)
Gravidity	
Mean	2.24
Parity	
Mean	0.53
Conception	
Spontaneous	94.2 (259)
Assisted	5.5 (15)
Not reported/Missing	0.3 (1)
Body Mass Index (kg/m^2^)	
Underweight (<18.5)	1.5 (4)
Normal weight (18.5–24.9)	37.5 (103)
Overweight (25.0–29.9)	30.5 (84)
Obese (30.0–39.9)	20 % (53)
Morbidly Obese (>40.0)	3.7 % (10)
Not reported/Missing	7.6 (21)
Current Smoking Status	
Smoker	13.5 (37)
Non-smoker	84.4 (232)
Not reported/Missing	2.2 (6)
Previous First Trimester Miscarriage	
Yes	28.2 (76)
No	71.7 (193)
Not reported/Missing	2.2 (6)
Gestation at booking	
Less than 12 weeks	20.7 (57)
12–20 weeks	63.6 (175)
More than 20 weeks	4 (11)
Not reported/Missing	12.7 (32)
Gestation at presentation with RFM	
28–31 weeks	13.1 (37)
31–34 weeks	2.7 (36)
34–37 weeks	20.6 (58)
37–40 weeks	40.0 (113)
More than 40 weeks	13.5 (38)

### Assessment and Management of RFM

In total, there were 282 presentations with RFM to the ED. As outlined in Table 1, 53.5 % (*n* = 151) women presented at more than 37 weeks’ gestation for assessment.

Table 2 indicates that the majority of women had a measurement of blood pressure (85.8 %; *n* = 242), and a CTG performed (97.9 %; *n* = 276) on presentation. Of the 276 CTGs performed, 20 (7.2 %) fulfilled criteria to classify the CTG as having “non-reassuring” features, of which 11 (55 %) women were delivered on this presentation. Amniotic fluid Index (AFI) assessment was performed in 69.9 % (*n* = 197) of women, with 12.4 % (*n* = 35) of women being referred for formal departmental ultrasound. Of patients having an AFI documented,, 17.3 % (*n* = 34) were classified as having a reduced AFI, and 41.1 % (*n* = 14) of these women delivered on this presentation.

**Table 2:** Investigations performed to investigate RFM

Parameter	Percentage % (*n*)
Blood Pressure	85.8 (242)
Symphyseal Fundal Height	85.1 (240)
Cardiotocograph	97.9 (276)
AFI measurement	69.9 (197)
Fetal Assessment Unit	12.4 (35)

Following presentation with RFM, 26.5 % (*n* = 73) of women were admitted to hospital for further monitoring and management.

### Pregnancy outcomes

Pregnancy outcomes of women with RFM and those in the control group are shown in Table 3. The mean gestation at delivery in both groups was similar. Those in the RFM group were less likely to have a spontaneous onset of labour (*p* = 0.0044), and more likely to undergo an induction of labour (IOL) than the control group (*p* = 0.0011). However, there was no statistically significant difference in the mode of delivery between all groups. The indications for IOL for the RFM cohort is demonstrated in Table 4.

**Table 3:** Pregnancy outcomes of RFM and control group

Outcome	RFM Group (275)	Control (265)
Gestation (weeks) at delivery range (mean)	28 + 2–42 + 0 (39 + 4)	26 + 5–41 + 6 (39 + 4)
Spontaneous Onset of Labour % (*n*)*	42.8 (115)	54.3 (144)
IOL % (*n*)*	42.4 (114)	27.9 (74)
Spontaneous Vaginal Delivery % (*n*)	46.3 (125)	53.2 (141)
Operative Vaginal Delivery % (*n*)	21.1 (57)	16.9 (45)
Caesarean Section Rate % (*n*)	32.6 (88)	29.8 (79)
Birth-weight Mean	3412.7 g	3442.5 g
Apgar Scores < 7 at 1 min % (*n*)	7 (18)	n/a
Apgar Scores < 7 at 5 min % (*n*)	1.6 (5)	1.9 (5)
NNU admission % (*n*)	10.6 (28)	7.2 (19)

*p < 0.05

**Table 4:** Indications for Induction of Labour for RFM Group (*n* = 114)

Indication	Number (%)
RFM	42 (36.8)
Post-dates	24 (21.1)
Pre-eclampsia/Pregnancy Induced Hypertension	12 (10.5)
Prolonged Rupture of Membranes	7 (6.1)
Fetal (e.g. Intrauterine growth retardation/Oligohydramnios)	15 (13.2)
Other (Social, Pelvic pain, NSAPH, macrosomia)	10 (8.7)
Maternal Medical (Obstetric Cholestasis, Venous Thromboembolic Disease, Gestational Diabetes	4 (3.5)

Of the 73 women admitted following assessment for RFM, 79.5 % (*n* = 58) women were delivered on presentation, with 19.1 % (*n* = 14) of these going into spontaneous labour. Caesarean Section (CS) was performed in 5.4 % (*n* = 4) of cases, with 54.7 % (*n* = 40) undergoing an IOL for RFM. Four (5.4 %) of these patients were less than 37 weeks’ gestation. Excluding women delivered on their presentation with RFM, 36.7 % (*n* = 101) presented in spontaneous labour, with 29.5 % (*n* = 81) having an IOL and 10.5 % (*n* = 29) having an elective CS. Of the total RFM group, 15.2 % (42/275) underwent IOL for the specific indication of RFM. Of all the RFM group, 46.3 % (*n* = 125) proceeded to have a spontaneous vaginal delivery, with 32.6 % (*n* = 88) being delivered by CS.

In the control group, 54.3 % (*n* = 144) women went into spontaneous labour, with 27.9 % (*n* = 74) having an IOL and 17.7 % (*n* = 47) having an elective CS. 53.2 % (*n* = 141) proceeded to a spontaneous vaginal delivery, with 29.8 % (*n* = 79) being delivered by emergency CS. There was no statistically significant difference between infant birth-weights or pre-term delivery rates in both groups.

## Discussion

This study presents a prospective review of women presenting with RFM in pregnancy, providing an examination of the management of their presentation and subsequent pregnancy outcome. Concurrently, in comparison to a control group, we demonstrated a higher rate of primiparous women presenting with RFM, with these women having a higher induction rate, but not significantly impacting CS rates or neonatal unit admission rates.

The stillbirth rate of this group of women presenting with RFM over 28 weeks’ gestation was 14.5 per 1000, which is significantly higher than the institutional corrected stillbirth rate (of women over 28 weeks’ gestation) of 2.9 per 1000 in 2013 [16]. This difference highlights the increased rate of intra-uterine fetal death of women presenting with RFM, and justifies the importance of evaluating and managing this problem in maternity units.

We demonstrate that the demographic breakdown of women presenting with RFM is varied. We illustrate that presentations with RFM tend to be associated with being a primigravida, but there is also a significant number of multiparous women presenting with RFM. The rates of primigravidae in our RFM group however were similar to the overall percentage of primigravidae in our hospital (38.5 %; *n* = 3143) in 2013 [16]. Despite those with RFM accounting for a small proportion of those assessed in the ED, over one quarter are admitted, which contrasts to other studies, which have a lower admission, but higher intervention rate [17]. This reflects the lack of consensus and guidelines for the management of RFM [18, 19]. A recent cross-sectional survery of maternity units also echoed this, finding guidelines from RFM were variable and frequently of low quality [20]. This high admission rate, and the subsequent delivery rate of our hospital implies that women admitted to our hospital with a complaint of RFM have up to an 80 % chance of being delivered. This also acknowledges that women with RFM have a higher iatrogenic delivery rate, and thus a higher induction rate than their non-RFM counterparts. There is no difference in CS rates between case and control groups despite increased IOL rates, which is echoed by other authors [21, 22].

This study includes a large number of women presenting with the complaint of RFM, examining the evaluation and management of these women, as well as assessing pregnancy outcomes. The prospective collection method of women presenting with RFM ensured inclusion of all women presenting to the ED of a large tertiary referral hospital, and allowed collection of data pertinent to their presentations. Comparing baseline demographics with a control group of women highlights how outcomes of pregnancy may be modified by this antenatal complaint. This study highlights the need for both local and national guidelines on the topic for RFM, to reduce the disparity of evaluation and management practices, and substantiates previous Irish prevalence rates of RFM [15].

As this is an observational study, the investigation and management strategies were at the discretion of the managing clinician. There is no clear consensus on what the optimum mode of assessment for RFM is, with guidelines recommending different modes and timing of assessments [10, 22]. Fetal growth and Doppler studies are routinely recommended in the presence of RFM [10, 23]; however this has not been shown to improve perinatal mortality rates [24]. In comparison to our study, some studies have demonstrated a 6.1 % admission rate following assessment [22] and higher CS rates for fetal compromise [23].

However, there are a number of limitations to this study. It was not possible to access full information from our control group, limiting comparison of data. Secondly, we were unable to collect all data for certain parameters in our control group, and despite interrogation of medical notes and delivery logbooks, some data could not be clarified retrospectively. Similarly, some data pertaining to the patient assessment in the ED was also not recorded in the notes, meaning that blood pressure measurements, and cardiotocography data could not be documented as performed, even though these details comprise an essential part of the initial assessment. Vital data such as gestation at delivery, Apgar scores, onset of labour and mode of delivery were all fully available, and therefore missing variables did not largely impact the analysis of the main outcomes measures. Finally, as there are no guidelines regarding the assessment and management of RFM, investigation and management decisions were based on individual experience rather than protocols.

Staff training in the area of RFM should emphasise acknowledging the importance of subjective maternal perception of RFM, and not place the focus solely on formal fetal counting methods, as has been emphasised over recent years [11, 12].

## Conclusions

RFM is a frequently occurring antenatal presentation, associated with poor perinatal outcomes. This study highlights its importance, and the need for continued research, education and training in the identification, investigation and management of RFM. Further research is needed to clarify optimum management strategies to optimise maternal and fetal outcome. RFM is an important part of risk assessment in antenatal care, and it is clear that hospitals should examine the prevalence and management of RFM within their services [22].

## Abbreviations

RFM, reduced fetal movements; CTG, cardiotocograph; ED, emergency department; AFI, amniotic fluid index; IOL, induction of labour; CS, caesarean section

## References

[1] Sergent F, Lefevre A. Verspyck E, Marpeau L. Decreased fetal movements in the third trimester: what to do? Gynécologie Obstétrique Fertilité. 2005;33:861–9. 10.1016/j.gyobfe.2005.07.041 16243568

[2] Efkarpidis S, Alexopoulos E, Kean L. Liu D, Fay T. Case–control study of factors associated with intrauterine fetal deaths”. Medscape General Med. 2000;6:53. PMC1395755 15266278

[3] Saastad E, Vangen S, Froen JF (2007). Suboptimal care in stillbirths - a retrospective audit study”. Acta Obstet Gynecol Scand.

[4] Fossen D, Silberg IE (1999). Perinatal deaths in the county of Ostfold 1989–97. Tidsskr Nor Laegeforen.

[5] Knight M, Tuffnell D, Kenyon S, Shakespeare J, Gray R, Kurinczuk JJ, on behalf of MBRRACE-UK (2015). Saving Lives, Improving Mothers’ Care - Surveillance of maternal deaths in the UK 2011–13 and lessons learned to inform maternity care from the UK and Ireland Confidential Enquiries into Maternal Deaths and Morbidity 2009–13.

[6] Mangesi L, Hofmeyr GJ, Smith V, Smyth RM (2015). Cochrane Collaboration. Fetal movement counting for assessment of fetal wellbeing. Cochrane Database Syst Rev.

[7] Saastad E, Winje BA, Pedersen S, Froen, J.F Fetal Movement counting Improved Identification of Fetal Growth Restriction and Perinatal Outcomes- a Multi-Centre, Randomized, Controlled Trial, Public Library of Science. PLoS One. 2011;6(12):e28482. 10.1371/journal.pone.0028482 PMC3244397 22205952

[8] Hofmeyr GJ, Novikova NM. Management of reported decreased fetal movements for improving pregnancy outcomes”, Database of Systematic Reviews 2012: 4, Art No: CD009148, DOI: 10.1002/14651858.CD009148.pub2 10.1002/14651858.CD009148.pub2 PMC4058897 22513971

[9] Preston S et al., Clinical management guideline for the management of women who report decreased fetal movements” Australian and New Zealand Stillbirth Alliance (ANZSA) 2010: http://www.stillbirthalliance.org.au/doc/FINAL%20DFM%20guideline%20Ed1V1%201_16Sept2010.pdf Accessed: 21 November 2015

[10] Royal College of Obstetrics and Gynaecology (RCOG) Reduced Fetal Movements. Green-top guideline No 57 2011: https://www.rcog.org.uk/globalassets/documents/guidelines/gtg_57.pdf. Accessed 21 November 2015

[11] Unterscheider J, Horgan R, O’Donoghue K, Greene R (2009). Reduced fetal movements. Obstetrician Gynaecol.

[12] Grant A, Elbourne D, Valentin L, Alexander S (1989). Routine formal fetal movement counting and risk of antepartum late death in normally formed singletons. Lancet.

[13] Tveit JV (2009). Reduction of late stillbirth with the introduction of fetal movement information and guidelines - a clinical quality improvement. BMC Pregnancy Childbirth.

[14] Winje BA et al., Interventions to enhance maternal awareness of decreased fetal movement: a systematic review. BJOG. DOI: 10.1111/1471-0528.13802 10.1111/1471-0528.13802 26629884

[15] Smith V, Begley C, Devane D (2014). Detection and management of decreased fetal movements in Ireland: a national survey of midwives’ and obstetricians’ practices. Midwifery.

[16] Cork University Maternity Hospital Annual Report. Wilton, Cork: Cork University Maternity Hospital; 2013.

[17] Awad NA, Jordan T, William M, Dan F (2014). Reduced fetal movements–management and outcome”. Am J Obstetrics Gynecol.

[18] Preboth M. ACOG guidelines on antepartum fetal surveillance”. Am College Obstetricians Gynecol Am Family Physician. 2000;62(5):1184, 1187-8. 10997537

[19] National Institute for Clinical Excellence (NICE) Antenatal Care. CG62 2008: https://www.nice.org.uk/Guidance/CG62 Accessed 21 November 2015

[20] Jokhan S (2015). Evaluation of the quality of guidelines for the management of reduced fetal movement. BMC Pregnancy Childbirth.

[21] O’Neill E, Thorpe J (2013). Antepartum Evaluation of the Fetus and Fetal Well Being”. Clin Obstetrics Gynecol.

[22] Skornick Rapaport A (2004). Perinatal outcome among women with reduced perception of fetal movements. Am J Obstetrics Gynecol.

[23] Froen JF (2001). A kick from within--fetal movement counting and the cancelled progress in antenatal care”. J Perinat Med.

[24] Daly N, Brennan D, Foley M, O’Herlihy C (2011). Cardiotocography as a predictor of fetal outcome in women presenting with reduced fetal movement”. Eur J Obstetrics Gynecol Reproduct Biol.

